# SIAH2 suppresses c‐JUN pathway by promoting the polyubiquitination and degradation of HBx in hepatocellular carcinoma

**DOI:** 10.1111/jcmm.18484

**Published:** 2024-06-06

**Authors:** Qinghe Hu, Zhiyi Liu, Yao Liu, Jie Qiu, Xue Zhang, Jun Sun, Bin Zhang, Hengliang Shi

**Affiliations:** ^1^ Institute of Digestive Diseases Xuzhou Medical University Xuzhou Jiangsu China; ^2^ Research Center of Digestive Diseases The Affiliated Hospital of Xuzhou Medical University Xuzhou Jiangsu China; ^3^ Department of General Surgery The Affiliated Hospital of Xuzhou Medical University Xuzhou Jiangsu China; ^4^ Central Laboratory The Affiliated Hospital of Xuzhou Medical University Xuzhou Jiangsu China

**Keywords:** c‐JUN, HBx, HCC, SIAH2, ubiquitination

## Abstract

As an important protein encoded by hepatitis B virus (HBV), HBV X protein (HBx) plays an important role in the development of hepatocellular carcinoma (HCC). It has been shown that seven in absentia homologue 1 (SIAH1) could regulates the degradation of HBx through the ubiquitin‐proteasome pathway. However, as a member of SIAH family, the regulatory effects of SIAH2 on HBx remain unclear. In this study, we first confirmed that SIAH2 could reduce the protein levels of HBx depending on its E3 ligase activity. Moreover, SIAH2 interacted with HBx and induced its K48‐linked polyubiquitination and proteasomal degradation. Furthermore, we provided evidence that SIAH2 inhibits HBx‐associated HCC cells proliferation by regulating HBx. In conclusion, our study identified a novel role for SIAH2 in promoting HBx degradation and SIAH2 exerts an inhibitory effect in the proliferation of HBx‐associated HCC through inducing the degradation of HBx. Our study provides a new idea for the targeted degradation of HBx and may have great huge significance into providing novel evidence for the targeted therapy of HBV‐infected HCC.

## 1 INTRODUCTION

Hepatocellular carcinoma (HCC) is a common malignant tumour of the digestive system, primarily caused by infection with hepatitis B virus (HBV), with its encoded envelope protein (S/Pre‐S), core protein (C/Pre‐C), polymerase (P) and X protein (HBx).^1^ HBx, is a multi‐functional regulatory protein crucial in HBV‐induced hepatocarcinogenesis, impacting various cellular activities such as cell proliferation, apoptosis, differentiation, drug resistance, transformation and DNA repair.^2^, ^3^, ^4^ Notably, HBx‐induced activation of the oncogene c‐JUN promotes abnormal proliferation and differentiation of liver cells, causing liver cell carcinogenesis.^4^, ^5^

c‐JUN, a member of the Jun family, acts as a downstream effector molecule of the MAPK signalling pathway, rapidly responding to external stimuli.^6^, ^7^ It holds essential biological roles in cell proliferation, differentiation and apoptosis.^8^ Genome‐wide expression analysis of human clinical HCC has demonstrated that c‐JUN plays a determinant role in oncogenic signalling pathways of HCC cases with poor prognosis.^9^ In a diethylnitrosamine‐induced (DEN) induced mouse HCC model, c‐JUN promoted the settlement of HCC through inhibition of P53,^10^ implying its proto‐oncogenic role in HCC development.

The SIAH (seven in absolute homologue) protein family comprises the mammalian homologue of the Drosophila SINA protein, and its human homologues include SIAH1, SIAH2 and SIAH3.^11^, ^12^ SIAH1 and SIAH2 coordinate ubiquitin‐mediated protein hydrolysis and regulate protein stability, assembly of protein complexes, and their subcellular localization by modifying and targeting multiple substrates, controlling cell function, growth, development and chemotherapy/radiation sensitivity of cancer cells.^13^, ^14^

Despite sharing 77% sequence similarity, SIAH1 and SIAH2 exhibit distinct substrates and varying affinities for their shared substrates.^15^ The mRNA and protein levels of SIAH1 exert tumour‐inhibitory effects and have been reported as downregulated in cancer.^16^, ^17^ In contrast, SIAH2 is widely expressed in different cell types and participates in the ubiquitination and degradation of multiple substrates, such as PHD3, HIPK2, p300, Tip60 and PRCAF, thereby regulating cellular processes, including proliferation, invasion, and migration.^18^, ^19^, ^20^ SIAH2 has been identified as a promoter of various human malignant tumours, including prostate cancer,^21^ lung cancer,^22^ gastric cancer^23^ and HCC.^24^ SIAH2 also exhibits tumour‐inhibitory effects in HCC by the degradation of oncoproteins through the proteasome pathway.^18^ Therefore, investigating the role of SIAH2 in the malignant progression of HCC might be determinant for the understanding of this pathology and its potential therapy.

In this study, we observed a SIAH2‐induced inhibition of c‐JUN by downregulation of HBx, reversing the proliferative effect of this pathway on liver cancer cells. Furthermore, we provide evidence of SIAH2 interaction with HBx and functioning as a ubiquitin ligase for HBx ubiquitination and degradation, consequently inhibiting its promoting effect on the proliferation of HCC cells. These findings regarding the SIAH2/HBx/c‐JUN axis offer new insights into HCC treatment.

## 2 MATERIALS AND METHODS

### 2.1 Plasmids and antibodies

Short hairpin RNA (shRNA) targeting SIAH2 (shSIAH2), HIS‐SIAH2 (WT and H99A/C102A), HA‐Ub (WT and mutant), FLAG‐HBx, and HBV‐1.3mer WT replicon plasmid were purchased from YouBio (Changsha, Hunan, China). Antibodies against SIAH2 (ab230523), HBx (ab157480), c‐Jun (ab40766), HIS (66005‐1‐Ig), FLAG (66008‐4‐Ig), HA (51064‐2‐AP), and‐actin (66009‐1‐Ig) were acquired from Abcam (Cambridge, UK). Phospho‐JUN (Ser73) Polyclonal antibody (28891‐1‐AP) was purchased from Proteintech (Wuhan, Hubei, China). HBcAg and HBsAg antibodies (bsm‐2000 M, bsm‐41,522 M) were purchased from Bioss (Beijing, China).

### 2.2 Transient transfection of siRNA and plasmid

SiRNA oligos for c‐JUN were purchased from GenePharma (Suzhou, China), with the following sequences:

Sense, 5′‐GGAAGCUGGAGAGAAUCGCTT‐3′;

Antisense, 5′‐GCGAUUCUCUCCAGCUUCCTT‐3′.

Cell transfection was performed using the Hieff Trans™ Liposomal Transfection Reagent (Yeasen, Shanghai, China) following manufacturer's instructions. Briefly, the plasmid and transfection reagent were mixed with the appropriate amount of serum‐free medium and then combined and mixed. After incubation at room temperature for 20 min, the mixture was added to the cells and the medium was replaced 6 h after transfection.

### 2.3 Cell culture

HEK293T, HepG2 (Non HBx associated virus HCC cells), and Hep3B (HBx associated virus in HCC cells) HCC cell lines were purchased from the Cell Bank (Chinese Academy of Sciences, Shanghai, China), and cultured in Minimum Essential medium (MEM) or Dulbecco's modified Eagle's medium (DMEM) (Yuanpei, Shanghai, China) supplemented with 10% fetal bovine serum (FBS, Gibco, Shanghai, China) at 37°C in an atmosphere of 5% CO_2_.

### 2.4 Lentivirus Construction

To silence SIAH2, three shRNA duplexes were designed as follows:

shSIAH2#1‐F:

gatccCCATGATGTGACTTTCGTAAATTCAAGAGATTTACGAAAGTCACATCATGGTTTTTTg.

shSIAH2#1‐R:

aattcAAAAAACCATGATGTGACTTTCGTAAATCTCTTGAATTTACGAAAGTCACATCATGGg.

shSIAH2#2‐F:

gatccGCCTACAGACTGGAGTTGAATTTCAAGAGAATTCAACTCCAGTCTGTAGGCTTTTTTg.

shSIAH2#2‐R:

aattcAAAAAAGCCTACAGACTGGAGTTGAATTCTCTTGAAATTCAACTCCAGTCTGTAGGCg.

shSIAH2#3‐F:

gatccACACAGCCATAGCACATCTTTTTCAAGAGAAAAGATGTGCTATGGCTGTGTTTTTTTg.

shSIAH2#3‐R:

aattcAAAAAAACACAGCCATAGCACATCTTTTCTCTTGAAAAAGATGTGCTATGGCTGTGTg.

To generate the lentiviruses, we co‐transfected HepG2 and Hep3B cells with the corresponding plasmids (Scramble or shSIAH2) and the helper plasmids (psPAX2 and pMD2G) using Hieff Trans™ Liposomal Transfection Reagent (Yeasen). For stable silencing of SIAH2, HepG2 and Hep3B cells were infected with scrambled or shSIAH2 viruses. Forty‐eight hours after infection, the cells were continuously cultured in a medium containing 2.5 μg/mL puromycin (Beyotime, Shanghai, China). Surviving cells were cultured in cell lines stably expressing scrambled shRNA or shSIAH2.

### 2.5 Western blotting

For western blotting, cells were lysed in radioimmunoprecipitation assay (RIPA) buffer supplemented with a protease inhibitor cocktail and centrifuged at 12,000×*g* at 4°C for 10 min. Equal amounts of protein were subjected to SDS‐PAGE and transferred onto a 0.45‐μm pore size PVDF membrane (Millipore, Billerica, MA, USA). After blocking with 3% bovine serum albumin (BSA; Yeasen), the membrane was incubated overnight with primary antibodies at 4°C and then with secondary antibodies for 1 h at room temperature. After washing the membranes with Tris‐buffered saline (TBST), the ECL Plus Western Blotting Substrate (Thermo Fisher Scientific, Waltham, MA, USA) was used to detect the protein bands, and a chemiluminescence detection system (Tanon, Shanghai, China) was used for visualization. Quantification of band intensity was conducted in ImageJ 1.8.0 (NIH, Bethesda, MD, USA).

### 2.6 5‐Ethynyl‐2′‐deoxyuridine (EdU) assay

To examine HCC cell proliferation, EdU incorporation assays were conducted using an EdU Assay Kit (RiboBio, Guangzhou, China). Briefly, cells were seeded in 96‐well plates at a density of 10,000 cells/well and cultured for 24 h. Cells were then incubated with 50 mM EdU at 37°C for 2 h and washed with PBS. Subsequently, cells were fixed with 4% paraformaldehyde in PBS for 30 min, and then permeabilized with 0.1% Triton X‐100 for 10 min. The cells were then washed twice with PBS for 5 min and treated with a 1× Apollo® reaction cocktail at room temperature for 30 min in the dark. Finally, nuclei were stained with 5 mg/mL Hoechst 33342 for 30 min and imaged using a fluorescence microscope (IX73; Olympus, Tokyo, Japan).

### 2.7 Plate colony formation

A cell suspension consisting of 400 cells in 5 mL of medium was seeded into a 60‐mm diameter culture dish for continuous culture until visible clones formed. Cells were then fixed with methanol and stained with a 0.05% crystal violet solution. After two washes with PBS, the plates were photographed using a digital camera. Positive colony formation, defined as colonies containing more than 50 cells, was confirmed by manual counting.

### 2.8 Cell counting Kit‐8 assay

Cell viability was assessed using the cell counting kit‐8 assay (CCK‐8, Dojindo, Japan). Briefly, cells were seeded in a 96‐well plate at a density of 5000 cells per well and cultured for 24 h. At the designated time points, 10 μL of CCK‐8 reagent was added to the cells and incubated at 37°C for 4 h, after which a SynergyMx MultiMode Microplate reader (Tecan, Switzerland) was used to detect the absorbance at 450 nm. Cell viability was calculated based on the absorbance values, following manufacturer's instructions.

### 2.9 Co‐immunoprecipitation (Co‐IP) assay

HEK293T, HepG2, and Hep3B cells were transfected with the indicated plasmids. After 24 h of transfection, MG132 added to the cells for an additional 6 h. The cells were then lysed in IP lysis buffer (1% Triton‐X‐100, 150 mM NaCl, 20 mM HEPES, 2 mM EDTA, 5 mM MgCl_2_, pH 7.4). Next, the extracted protein supernatant was incubated overnight at 4°C with the specific antibodies (HIS, 1:100; FLAG, 1:200;SIAH2, 1:200; HBx, 1:200), followed by co‐incubation with protein A/G‐Magnetic Beads (MCE, Shanghai, China). The magnetic beads were washed thrice with PBS‐T (1 × PBS + 0.5% Triton X‐100, pH 7.4) and boiled in an SDS sample buffer for 10 min. Immunoprecipitation was performed using western blotting with the indicated antibodies.

### 2.10 Ubiquitination assay

HA‐Ub, FLAG‐HBx, HIS‐SIAH2 and corresponding plasmids (K6R, K11R, K27R, K29R, K33R, K48R and K63R) were separately co‐expressed in HepG2 and Hep3B cells and incubated with a proteasome inhibitor (MG132) for 6 h before collection. The collected cells were lysed with Triton X‐100‐based lysis buffer (1% Triton X‐100; 150 mM NaCl; 20 mM 4‐(2‐hydroxyethyl)‐1‐piperazineethanesulfonic acid (HEPES), pH 7.4; 2 mM EDTA, pH 8.0; 5 mM MgCl_2_) supplemented with protease inhibitors, followed by centrifugation at 12,000×*g* at 4°C. A 1 mg/mL protein solution was prepared, and 20 μL of magnetic beads were added and incubated at 4°C for 1 h to remove nonspecific binding. The supernatant was then mixed with an appropriate amount of primary antibody (FLAG, HBx.) and incubated overnight at 4°C. After adding 40 μL of magnetic beads, the application was continued for 4 h at 4°C. The magnetic beads were washed 5 times with pre‐chilled 1× TBS, and boiled in an SDS sample buffer, which was then analysed using western blotting.

### 2.11 Data analysis

All experiments were repeated a minimum of three times independently, and representative results are shown. Results are shown as the mean ± standard deviation. GraphPad Prism software (GraphPad software Inc., San Diego, CA, USA) was used for statistical analysis. Differences between two groups were analysed using the Student's *t*‐test. Statistical significance was set at *p* < 0.05.

## 3 RESULTS

### 3.1 HBx promotes HCC cells proliferation through regulation of c‐JUN

**FIGURE 1:**
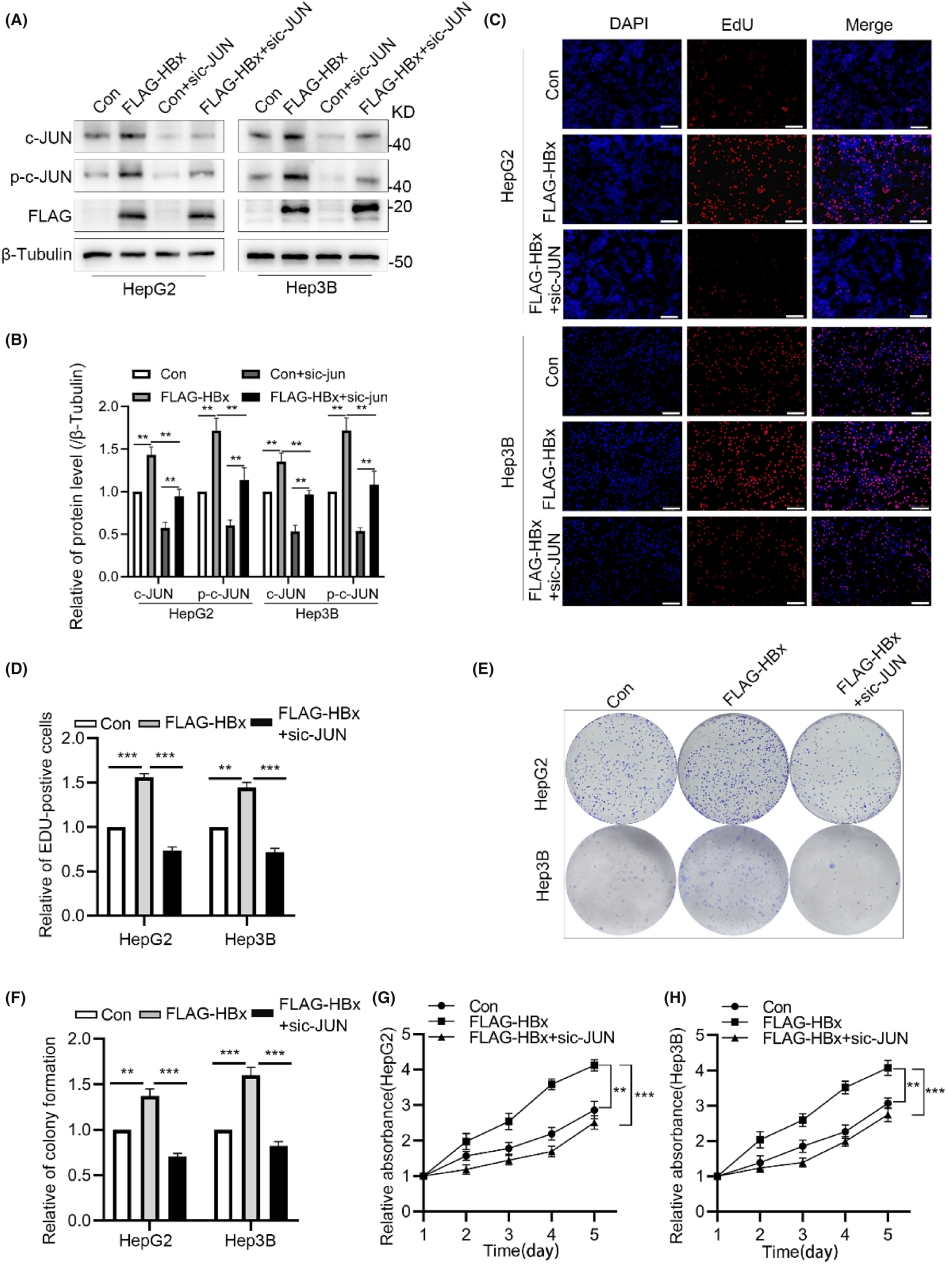
HBx promotes the proliferation of HCC cells through upregulation of c‐JUN. (A, B) Western blot depicting protein levels of c‐JUN, p‐c‐JUN and FLAG‐HBx in HepG2 and Hep3B cells. (C, D) EdU staining revealing a significant increase in the proportion of EdU‐positive cells after HBx overexpression, whereas downregulation of c‐JUN expression inhibits this effect. Scale bar: 200 μM. (E, F) Cell clone formation assay demonstrating that overexpression of HBx enhances the proliferation of HCC cells, whereas downregulation of c‐JUN suppresses this effect. (G, H) CCK‐8 indicating that overexpression of HBx enhances the proliferation of HCC cells, while downregulation of c‐JUN expression counteracts this effect. **p < 0.01, ***p < 0.001.

The role of c‐JUN in counteracting the cell growth‐promoting effect induced by hepatitis B virus X protein (HBx) in HCC has been highlighted through inhibition of c‐JUN N‐terminal kinase.^25^ To investigate the involvement of c‐JUN in HBx‐induced HCC cell proliferation, we overexpressed FLAG‐tagged HBx or co‐transfected it with c‐JUN siRNA in HepG2 and Hep3B cells. Western blotting confirmed that the overexpression of HBx significantly elevated the protein levels of c‐JUN and phosphorylated c‐JUN (Figure 1A,B). The effect of HBx on the proliferation of HCC cells was assessed through EdU incorporation and colony formation assays. The overexpression of HBx increased the number EdU‐positive cells, whereas c‐JUN silencing inhibited this effect (Figure 1C,D). Similarly, colony formation ability was enhanced by overexpression of HBx, and this effect was reversed upon c‐JUN silencing (Figure 1E,F). Furthermore, the CCK‐8 assays (pertaining to cell viability) yielded results consistent with to those of the EdU and colony formation assays (Figure 1G,H). These findings suggest that HBx promotes the proliferation of HCC cells by positively regulating c‐Jun expression.

### 3.2 SIAH2 regulates the stability of HBx through its E3 activity

**FIGURE 2:**
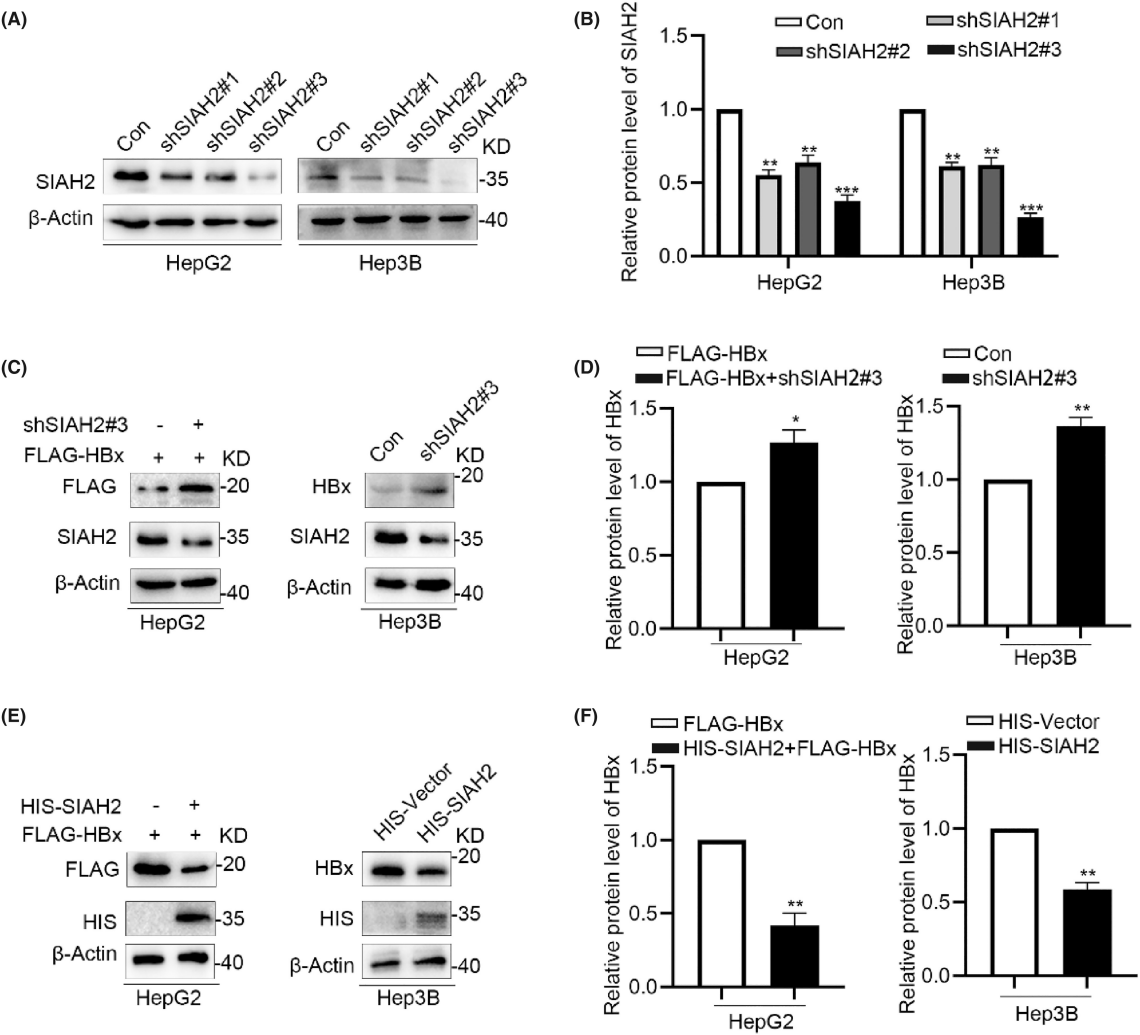
SIAH2 negatively regulates the expression of HBx in liver cancer cells. (A, B) Western blot validation of the effective downregulation effect on SIAH2 by the three specific silencing vectors (shSIAH2#1, shSIAH2#2 and shSIAH2#3) in HepG2 and Hep3B cells. shSIAH2#3 showed the most effective downregulation effect. (C, D) Western blot depicting that transfection of shSIAH2#3 in HepG2 and Hep3B cells increases HBx protein levels. (E, F) Western blot validation indicating that overexpression of SIAH2 in HepG2 and Hep3B cells downregulates HBx protein levels. *p < 0.05, **p < 0.01, ***p < 0.001.

Considering that SIAH1 promotes the ubiquitination and degradation of HBx,^26^ we explored whether SIAH2, as a member of the SIAH family with high sequence similarity with SIAH1,^27^ also regulates HBx expression. To address this question, we evaluated HBx protein levels in HCC cells following SIAH2 downregulation or upregulation. SIAH2 knockdown was achieved using three shRNAs targeting SIAH2 (shSIAH2#1, shSIAH2#2 and shSIAH2#3), and the most efficient silencing was observed with shSIAH2#3 (Figure 2A,B). Subsequently, we analysed HBx protein levels in HCC cells upon SIAH2 silencing, and the results showed that SIAH2 knockdown increased both exogenous or exogenous HBx in HepG2 cells and endogenous HBx in Hep3B cells (Figure 2C,D). To further investigate the regulatory effect of SIAH2 on HBx, we transiently transfected HIS‐tagged SIAH2 cDNA in HepG2 and Hep3B cells to achieve a gain‐of‐function. Conversely, overexpressing SIAH2 decreased the protein levels of exogenous HBx in HepG2 cells and endogenous HBx in Hep3B cells (Figure 2E,F). Furthermore, we also explored the effect of HBV replication on the expression level of SIAH2, we transfected HBV‐1.3mer WT replicon plasmid, and Western blot assay showed no significant change on the expression of SIAH2 (Figure S1A). On the other hand, we transfected Flag‐tagged HBx in HepG2 cells, and the result indicated that HBx had no effect on the expression of SIAH2 (Figure S1B). The above results showed that SIAH2 could downregulate the HBx protein level, and HBx has no effect on SIAH2 expression.

**FIGURE 3:**
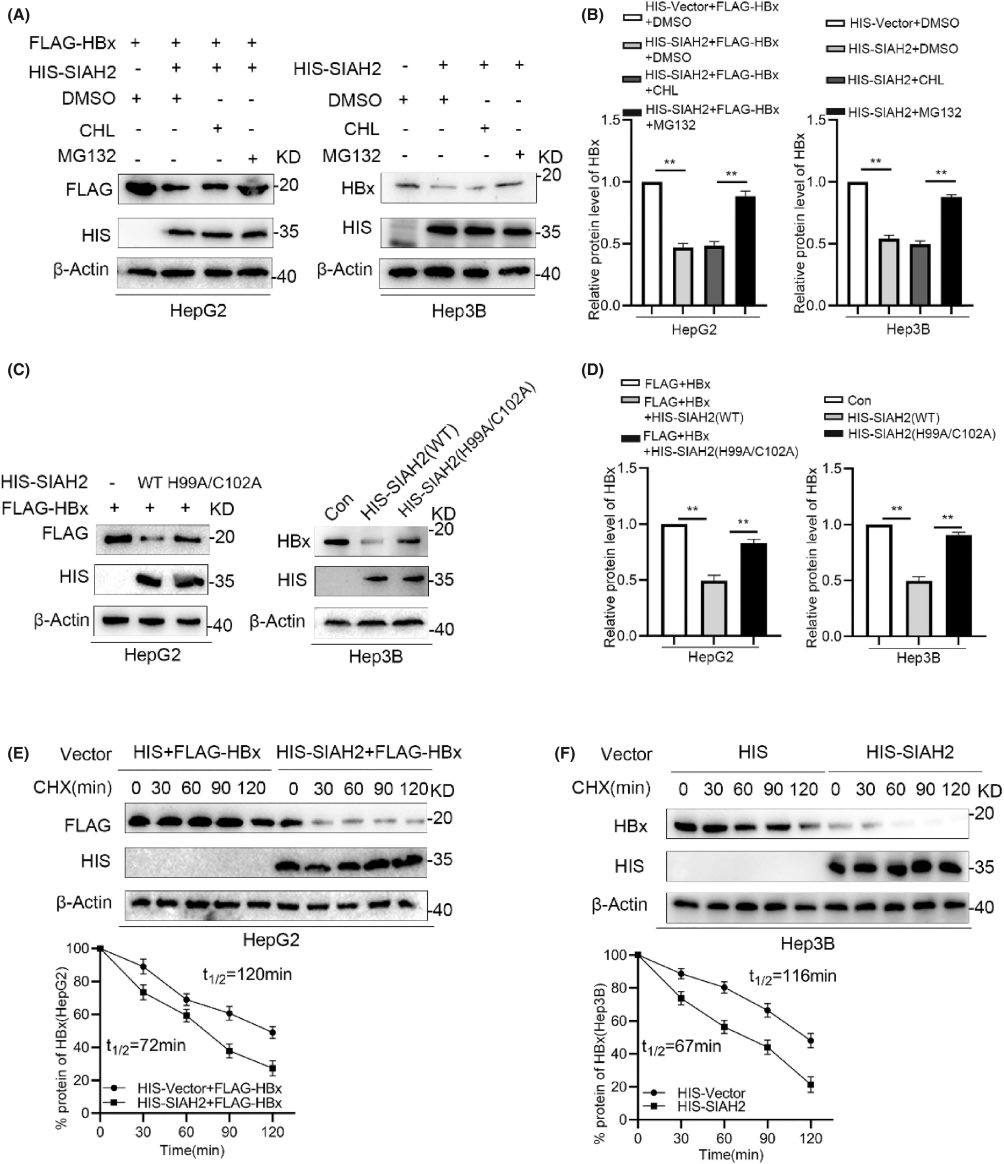
SIAH2 reduces the protein stability of HBx. (A, B) Western Blot demonstrating the reversion of the negative regulatory effect of SIAH2 on HBx in HepG2 and Hep3B cells by MG132 treatment. (C, D) Western blot indicating that the downregulation effect of HIS‐SIAH2 (H99A/C102A) transfection on HBx is weaker in HepG2 cells and Hep3B cells compared to the HIS‐SIAH2 (WT) transfection group. (E) CHX chase assay shows that SIAH2 disrupts the exogenous protein stability of HBx in HepG2 cells during CHX chase for 0, 30, 60, 90, and 120 min. (F) CHX chase assay demonstrates that SIAH2 disrupts the endogenous protein stability of HBx in Hep3B cells during CHX chase for 0, 30, 60, 90, and 120 min. **p < 0.01.

SIAH2 regulates several downstream substrates via the proteasomal pathway and decreases protein stability.^28^ To understand how SIAH2 regulates HBx, we treated SIAH2‐overexpressing cells with the proteasome inhibitor MG‐132 and the lysosome inhibitor chloroquine (CHL). MG‐132, but not chloroquine, blocked the degradation of HBx in cells with overexpression of SIAH2 (Figure 3A,B). When His^99^ and Cys^102^ in the RING domain are converted to alanine (H99A/C102A), SIAH2 has no E3 ligase activity.^29^ To determine the importance of SIAH2's E3 ligase activity in the downregulation of HBx, we generated a SIAH2‐RING mutant (H99A/C102A) lacking E3 ligase activity. Compared to wild‐type SIAH2 (SIAH2‐WT), the SIAH2‐RING mutant largely lost its ability to decrease HBx protein levels (Figure 3C,D). Furthermore, we treated the cells with the protein synthesis inhibitor cycloheximide (CHX) to determine the stability of HBx upon SIAH2 overexpression and observed a loss of stability of HBx following SIAH2 overexpression (Figure 3E,F). Collectively, these results suggest that SIAH2 destabilizes HBx via the proteasomal pathway in a manner dependent on its E3 activity.

### 3.3 SIAH2 inhibits HBx‐associated HCC cells proliferation by promoting Lys48‐linked polyubiquitination of HBx

**FIGURE 4:**
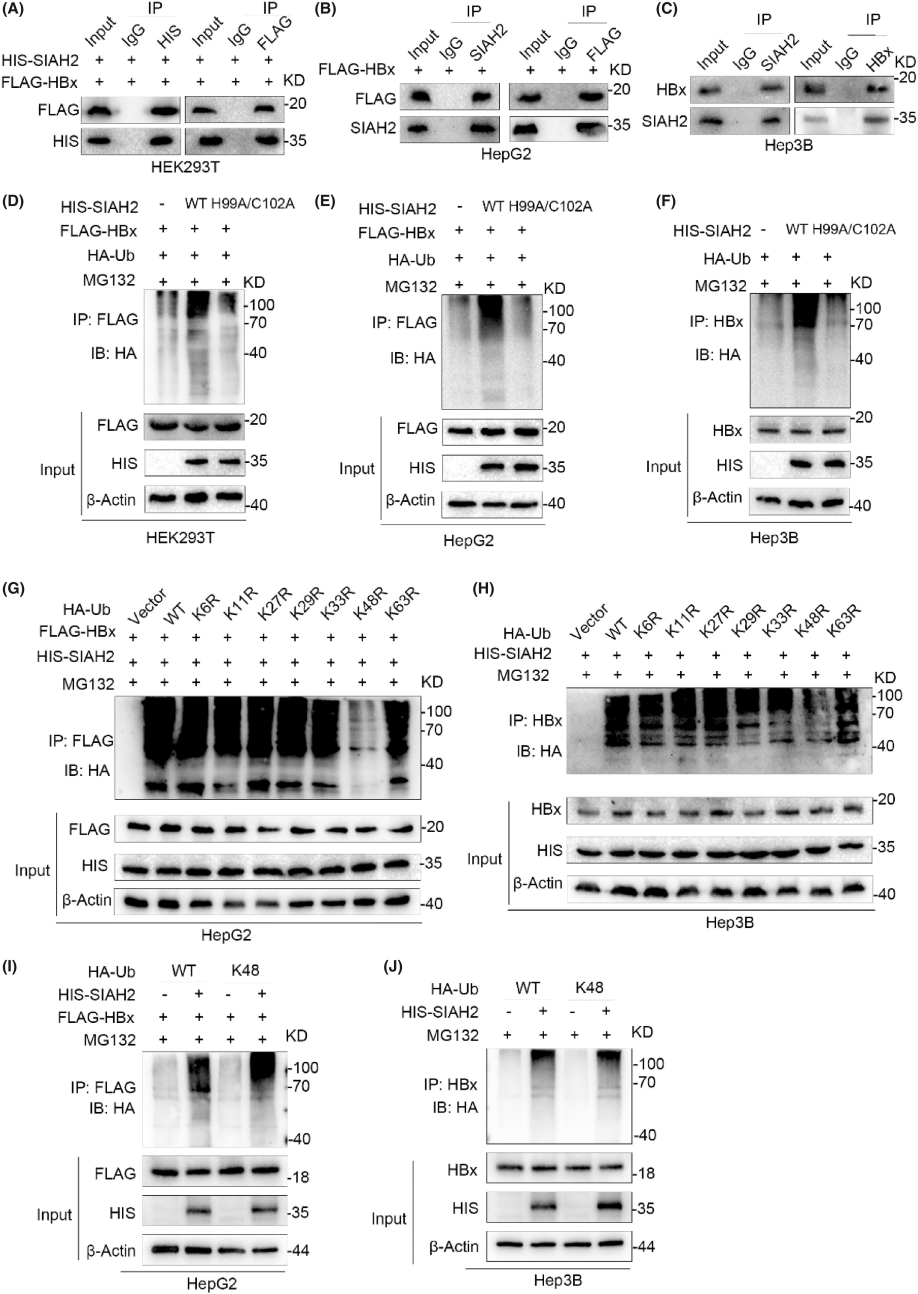
SIAH2 mediates K48‐linked polyubiquitination of HBx. (A) Co‐immunoprecipitation (Co‐IP) validation shows the binding of HIS‐SIAH2 and FLAG‐HBx in HEK293T cells. (B) Co‐IP validation reveals the presence of SIAH2 and FLAG‐HBx in the same complex in HepG2 cells. (C) Co‐IP validation demonstrates the interaction between SIAH2 and HBx in Hep3B cells. (D) Western blot analysis demonstrating that the ubiquitination level of HBx in HEK293T cells transfected with HIS‐SIAH2 (H99A/C102A) is lower than that in HIS‐SIAH2 (WT); (E) Western blot analysis revealing that overexpression of SIAH2 in HepG2 cells increases the ubiquitination levels of HBx, while transfection with HIS‐SIAH2 (H99A/C102A) decreases it. (F) Ubiquitination levels of endogenous HBx in Hep3B cells. (G) Transfection with ubiquitin mutants (K6R, K11R, K27R, K29R, K33R, K48R and K63R) in HepG2 cells. Blocking K48‐linked ubiquitin chain decreases the ubiquitination levels of exogenous HBx. (H) Transfection with ubiquitin mutants (K6R, K11R, K27R, K29R, K33R, K48R and K63R) in Hep3B cells. Blocking K48‐linked ubiquitin chain decreases the ubiquitination levels of endogenous HBx. (I, J) Transfection with HA‐Ub‐WT or HA‐Ub‐K48 in HepG2 and Hep3B cells, and overexpression of SIAH2 can increase the K48‐linked polyubiquitination of HBx.

To explore the regulatory mechanism of SIAH2 on HBx, first, we tested the interaction relationship between SIAH2 and HBx. HIS‐tagged SIAH2 interacted with FLAG‐tagged HBx in HEK293T cells, whereas endogenous SIAH2 interacted with both FLAG‐HBx and endogenous HBx in HCC cells (Figure 4A–C). In addition, we also detected the interaction between SIAH2 and other HBV proteins. The results showed that SIAH2 only interacted with HBx, but not HBc and HBs, which is similar to SIAH1 (Figure S1C). To investigate the effects of SIAH2 on HBx ubiquitination, we performed ubiquitination assays. Cells transfected with HIS‐SIAH2 exhibited significantly increased HBx ubiquitination, whereas the SIAH2‐RING mutant reduced the ability of SIAH2 to ubiquitinate HBx in both HEK293T and HCC cells (Figure 4D–F). Ubiquitination in the proteasome pathway is mainly mediated by seven lysine residues: K6, K11, K27, K29, K33, K48, and K63.^30^ To verify the form of SIAH2 mediated ubiquitination of HBx, HCC cells were co‐transfected with HIS‐SIAH2 and WT or mutant HA‐Ub vectors, HBx was co‐precipitated with anti‐FLAG or HBx antibodies, and the levels of exogenous and endogenous HBx ubiquitination were determined by western blotting with an HA antibody. HBx polyubiquitination was markedly blocked when the lysine at position 48 of Ub was mutated to alanine (K48R), indicating that SIAH2‐mediated HBx ubiquitination was primarily initiated by K48‐linked ubiquitin chains, which are recognized by the 26S proteasome (Figure 4G,H). To further confirm that SIAH2 mediates K48‐linked polyubiquitination of HBx, we used a Ub K48‐only plasmid, in which only K48 is encoded and other lysine are mutated to arginine. Then, the results showed that SIAH2 could promote the K48‐linked polyubiquitination of HBx (Figure 4I,J).

**FIGURE 5:**
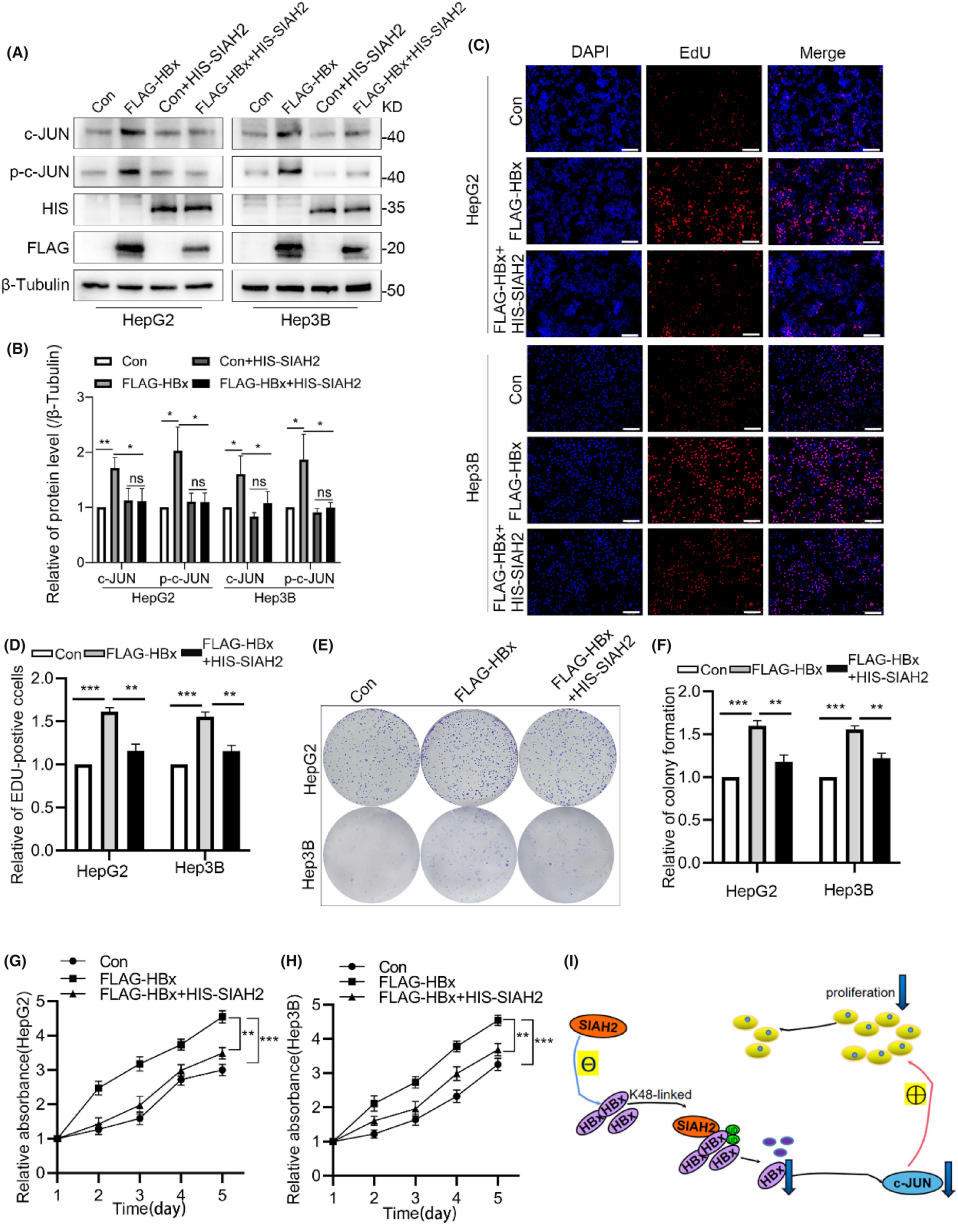
SIAH2 regulates HBx inhibition of c‐JUN affecting the proliferation of liver cancer cells. (A, B) Western blot demonstrating that the overexpression of SIAH2 in HepG2 and Hep3B cells downregulates of HBx and c‐JUN expression. (C, D) EdU assay of HepG2 and Hep3B cells transfected with FLAG‐HBx, FLAG‐HBx + HIS‐SIAH2 showing that the proliferation‐promoting effect of HBx is weakened upon overexpression of SIAH2; (E, F) Cell colony formation assay of HepG2 cells and Hep3B cells with FLAG‐HBx and FLAG‐HBx + HIS‐SIAH2 reveal that overexpression of HBx enhances the proliferation of liver cancer, while the co‐overexpression of HBx and SIAH2 hinders this proliferation. (G, H) CCK‐8 assay after transfection of HepG2 cells and Hep3B cells with FLAG‐HBx and FLAG‐HBx + HIS‐SIAH2, respectively. (I) Proposed mechanism of ubiquitination and degradation of HBx by SIAH2 and its impact on the proliferation of liver cancer cells. *p < 0.05, **p < 0.01, ***p < 0.001.

To determine whether SIAH2 affected the promotion of HCC proliferation caused by HBx overexpression, we performed rescue experiments by overexpressing HIS‐SIAH2 in HBx‐upregulated cells. Overexpression of SIAH2 effectively decreased the protein levels of c‐JUN and p‐c‐JUN, which were upregulated by HBx (Figure 5A,B), and subsequently inhibited cell proliferation induced by HBx overexpression (Figure 5C–H). These results demonstrate that SIAH2 counteracts the effect of HBx in the promotion of HCC cell proliferation.

Based on these findings, we propose a model diagram, as shown in Figure 5I, where in liver cancer cells infected with HBV, SIAH2 interacts with HBx and negatively regulates it through the ubiquitin‐proteasome pathway, leading to reduced stability of HBx. During this process, SIAH2 predominantly regulates the proteasomal degradation of HBx by mediating its K48‐linked ubiquitination. Overall, this indicates that SIAH2 effectively reverses the proliferative effect of the HBx/c‐JUN axis, highlighting its crucial role in inhibiting HCC proliferation.

## 4 DISCUSSION

HCC remains a significant global health concern and is a leading cause of cancer‐related mortality.^31^ Despite advancements in diagnosis and treatment over the past decade, the prognosis for advanced HCC remains poor.^32^ HBV infection a major contributor to HCC development.^33^ As a multi‐functional regulatory protein of HBV expression, HBx is involved in the pathogenesis and carcinogenesis of the virus.^34^ HBx has limited expression in early stages of infection and is observed in 20%–50% of HCC cases.^35^ HBx transactivates various transcription factors, including activating transcription factor 2 (ATF2),^36^ activating protein 1 (AP‐1)^37^ and nuclear factor kappa B (NF‐κB).^38^ Additionally, as a transcription factor, HBx stimulates the expression of proto‐oncogenes, controlling proliferation, transformation, apoptosis, and DNA repair in HCC cells.^39^ Furthermore, c‐JUN has been extensively studied as an important proto‐oncogene involved in the occurrence and development of HCC.^40^, ^41^ Our study confirmed that HBx promotes the proliferation of HCC cells by positively regulating c‐Jun, thereby uncovering a novel regulatory mechanism for c‐JUN.

Ubiquitination is an important post‐translational modification that promotes intracellular protein degradation via the proteasome multiprotease complex.^42^, ^43^ The stability of HBx is influenced by ubiquitination modifications.^44^ Several ubiquitin ligases, including TRIM21,^45^ NEDD4^46^ and SIAH1^26^ have been identified as regulators of the ubiquitination and degradation of HBx. Here, we explored the involvement of SIAH2, a member of the SIAH family with structural and functional similarity to SIAH1,^47^, ^48^ in the regulation of HBx. Our results demonstrated that SIAH2 promotes the ubiquitination and subsequent proteasomal degradation of HBx, leading to a decrease in HBx protein levels. Importantly, we observed that SIAH2 also inhibits HCC cell proliferation induced by HBx overexpression, highlighting SIAH2 as a novel ubiquitin ligase regulating HBx.

Ubiquitination within the proteasomal pathway is mediated by seven lysine residues, namely K6, K11, K27, K29, K33, K48 and K63.^49^ These multi‐ubiquitin chains possess different topological structures, generating create complex ubiquitin codes that govern a myriad of biological functions.^30^ This study provides novel insights by defining K48‐linked chains as the key initiators of HBx polyubiquitination mediated by SIAH2. This finding holds potential for advancing research into canonical protein ubiquitination.

In conclusion, we identified HBx as a novel substrate of SIAH2 and shed light on the regulatory mechanism involving SIAH2 in HBx degradation. The downregulation of HBx expression by SIAH2 leads to the inhibition of HCC cell proliferation by modulation of c‐JUN. These findings offer new perspectives on the molecular mechanisms governing HBx and potential molecular targeted therapies for HBx‐associated HCC. Although we confirmed that SIAH2 could inhibit HCC cells proliferation through promoting the K48‐linked polyubiquitination and degradation of HBx, however, SIAH2 has also been reported to exhibit both promotional and inhibitory roles in HCC cells,^24^, ^50^ which may be influenced by factors such as the origin of the different HCC cell lines or the localization of SIAH2 within the cells. Due to the specificity of HBV infection, there may be a more complex mechanism by which SIAH2 regulates HBx in HCC. Unfortunately, our study lacks a model of HBV infection to reveal this regulatory relationship more fully. We believe that more in‐depth studies are needed for uncovering the complex mechanisms of HBV‐associated HCC.

## AUTHOR CONTRIBUTIONS

**Qinghe Hu:** Conceptualization (equal); data curation (lead); formal analysis (lead); methodology (equal); resources (equal); software (equal); validation (lead); visualization (lead); writing – original draft (lead); writing – review and editing (supporting). **Zhiyi Liu:** Conceptualization (lead); data curation (equal); formal analysis (equal); funding acquisition (lead); resources (equal); supervision (lead); visualization (equal); writing – review and editing (lead). **Yao Liu:** Conceptualization (supporting); data curation (equal); formal analysis (equal); methodology (equal); visualization (equal). **Jie Qiu:** Formal analysis (supporting); software (supporting); validation (supporting). **Xue Zhang:** Formal analysis (supporting); validation (supporting). **Jun Sun:** Formal analysis (supporting). **Bin Zhang:** Conceptualization (supporting); funding acquisition (lead); project administration (equal); supervision (lead). **Hengliang Shi:** Conceptualization (lead); funding acquisition (lead); project administration (lead); supervision (lead); writing – review and editing (lead).

## FUNDING INFORMATION

The work was supported by the Young Science and Technology Innovation Team of Xuzhou Medical University Key Research and Development Plan of Jiangsu Province [TD202006], Basic Research Program of Jiangsu Province [BK20231166], Jiangsu Provincial Commission of Health and Family Planning [M2020082].

## CONFLICT OF INTEREST STATEMENT

The authors declare that there is no conflict of interest between them in this study.

## INFORMED CONSENT STATEMENT

The HCC patients signed an internal regulatory document stating that the remaining samples could be used for a retrospective academic study without other informed consent.

## INSTITUTIONAL REVIEW BOARD STATEMENT

The study was performed according to the principles containd in the Declaration of Helsinki (2013). The Ethics Research Committee of the Affiliated Hospital of Xuzhou Medical University approved this study (Approval Number XYFY2019‐KL129‐01).

## Supporting information


Figure S1.

## Data Availability

The manuscript includes all datasets which support the conclusions of this paper.

## References

[1] McGlynn KA , Petrick JL , El‐Serag HB . Epidemiology of hepatocellular carcinoma. Hepatology. 2021;73 Suppl 1(Suppl. 1):4‐13. 10.1002/hep.31288 PMC7577946 32319693

[2] Sartorius K , An P , Winkler C , et al. The epigenetic modulation of cancer and immune pathways in hepatitis B virus‐associated hepatocellular carcinoma: the influence of HBx and miRNA dysregulation. Front Immunol. 2021;12:661204. 33995383 10.3389/fimmu.2021.661204 PMC8117219

[3] Zhou Q , Yan L , Xu B , et al. Screening of the HBx transactivation domain interacting proteins and the function of interactor Pin1 in HBV replication. Sci Rep. 2021;11(1):14176. 34238995 10.1038/s41598-021-93584-z PMC8266847

[4] You H , Yuan D , Li Q , et al. Hepatitis B virus X protein increases LASP1 SUMOylation to stabilize HER2 and facilitate hepatocarcinogenesis. Int J Biol Macromol. 2023;226:996‐1009. 36473530 10.1016/j.ijbiomac.2022.11.312

[5] Harputluoglu M , Carr BI . Hepatitis B before and after hepatocellular carcinoma. J Gastrointest Cancer. 2021;52(4):1206‐1210. 34762265 10.1007/s12029-021-00745-4

[6] Jadhav SP , Kamath SP , Choolani M , Lu J , Dheen ST . microRNA‐200b modulates microglia‐mediated neuroinflammation via the cJun/MAPK pathway. J Neurochem. 2014;130(3):388‐401. 24749688 10.1111/jnc.12731

[7] Dong Y , Xu W , Li Y , et al. Inhibition of the MAPK/c‐Jun‐EGR1 pathway decreases photoreceptor cell death in the rd1 mouse model for inherited retinal degeneration. Int J Mol Sci. 2022;23(23):14600. 36498926 10.3390/ijms232314600 PMC9740268

[8] Atsaves V , Zhang R , Ruder D , et al. Constitutive control of AKT1 gene expression by JUNB/CJUN in ALK+ anaplastic large‐cell lymphoma: a novel crosstalk mechanism. Leukemia. 2015;29(11):2162‐2172. 25987255 10.1038/leu.2015.127 PMC4633353

[9] Xiong DD , Dang YW , Lin P , et al. A circRNA‐miRNA‐mRNA network identification for exploring underlying pathogenesis and therapy strategy of hepatocellular carcinoma. J Transl Med. 2018;16(1):220. 30092792 10.1186/s12967-018-1593-5 PMC6085698

[10] Bitton‐Worms K , Pikarsky E , Aronheim A . The AP‐1 repressor protein, JDP2, potentiates hepatocellular carcinoma in mice. Mol Cancer. 2010;9:54. 20214788 10.1186/1476-4598-9-54 PMC2841123

[11] Weiskirchen S , Tag CG , Sauer‐Lehnen S , Tacke F , Weiskirchen R . Isolation and culture of primary murine hepatic stellate cells. Methods Mol Biol. 2017;1627:165‐191. 28836201 10.1007/978-1-4939-7113-8_11

[12] Zhang Q , Wang Z , Hou F , et al. The substrate binding domains of human SIAH E3 ubiquitin ligases are now crystal clear. Biochim Biophys Acta Gen Subj. 2017;1861(1 Pt A):3095‐3105. 27776223 10.1016/j.bbagen.2016.10.019

[13] Stebbins JL , Santelli E , Feng Y , et al. Structure‐based design of covalent Siah inhibitors. Chem Biol. 2013;20(8):973‐982. 23891150 10.1016/j.chembiol.2013.06.008 PMC3763817

[14] van Wijk SJ , de Vries SJ , Kemmeren P , et al. A comprehensive framework of E2‐RING E3 interactions of the human ubiquitin‐proteasome system. Mol Syst Biol. 2009;5:295. 19690564 10.1038/msb.2009.55 PMC2736652

[15] Della NG , Senior PV , Bowtell DD . Isolation and characterisation of murine homologues of the drosophila seven in absentia gene (sina). Development. 1993;117(4):1333‐1343. 8404535 10.1242/dev.117.4.1333

[16] Yoshibayashi H , Okabe H , Satoh S , et al. SIAH1 causes growth arrest and apoptosis in hepatoma cells through beta‐catenin degradation‐dependent and ‐independent mechanisms. Oncol Rep. 2007;17(3):549‐556. 17273732

[17] Telerman A , Amson R . The molecular programme of tumour reversion: the steps beyond malignant transformation. Nat Rev Cancer. 2009;9(3):206‐216. 19180095 10.1038/nrc2589

[18] Li K , Li J , Ye M , Jin X . The role of Siah2 in tumorigenesis and cancer therapy. Gene. 2022;809:146028. 34687788 10.1016/j.gene.2021.146028

[19] Zakaria S , Elsebaey S , Allam S , El‐Sisi A . Modulating the Siah2‐PHD3‐HIF1α axis and/or autophagy potentially retard colon cancer proliferation possibly, due to the damping of colon cancer stem cells. Biomed Pharmacother. 2022;154:113562. 35994813 10.1016/j.biopha.2022.113562

[20] Schmitz ML , Dreute J , Pfisterer M , Günther S , Kracht M , Chillappagari S . SIAH ubiquitin E3 ligases as modulators of inflammatory gene expression. Heliyon. 2022;8(3):e09029. 35284677 10.1016/j.heliyon.2022.e09029 PMC8904615

[21] Qi J , Tripathi M , Mishra R , et al. The E3 ubiquitin ligase Siah2 contributes to castration‐resistant prostate cancer by regulation of androgen receptor transcriptional activity. Cancer Cell. 2013;23(3):332‐346. 23518348 10.1016/j.ccr.2013.02.016 PMC3750989

[22] Ahmed AU , Schmidt RL , Park CH , et al. Effect of disrupting seven‐in‐absentia homolog 2 function on lung cancer cell growth. J Natl Cancer Inst. 2008;100(22):1606‐1629. 19001609 10.1093/jnci/djn365 PMC2720765

[23] Dixit P , Kokate SB , Poirah I , et al. *Helicobacter pylori*‐induced gastric cancer is orchestrated by MRCKβ‐mediated Siah2 phosphorylation. J Biomed Sci. 2021;28(1):12. 33536006 10.1186/s12929-021-00710-0 PMC7856738

[24] Malz M , Aulmann A , Samarin J , et al. Nuclear accumulation of seven in absentia homologue‐2 supports motility and proliferation of liver cancer cells. Int J Cancer. 2012;131(9):2016‐2026. 22323152 10.1002/ijc.27473

[25] Wu YH , Ai X , Liu FY , Liang HF , Zhang BX , Chen XP . c‐Jun N‐terminal kinase inhibitor favors transforming growth factor‐β to antagonize hepatitis B virus X protein‐induced cell growth promotion in hepatocellular carcinoma. Mol Med Rep. 2016;13(2):1345‐1352. 26648552 10.3892/mmr.2015.4644 PMC4732859

[26] Zhao J , Wang C , Wang J , et al. E3 ubiquitin ligase Siah‐1 facilitates poly‐ubiquitylation and proteasomal degradation of the hepatitis B viral X protein. FEBS Lett. 2011;585(19):2943‐2950. 21878328 10.1016/j.febslet.2011.08.015

[27] Hu G , Chung YL , Glover T , Valentine V , Look AT , Fearon ER . Characterization of human homologs of the drosophila seven in absentia (sina) gene. Genomics. 1997;46(1):103‐111. 9403064 10.1006/geno.1997.4997

[28] DeBruyne JP , Baggs JE , Sato TK , Hogenesch JB . Ubiquitin ligase Siah2 regulates RevErbα degradation and the mammalian circadian clock. Proc Natl Acad Sci USA. 2015;112(40):12420‐12425. 26392558 10.1073/pnas.1501204112 PMC4603519

[29] Habelhah H , Frew IJ , Laine A , et al. Stress‐induced decrease in TRAF2 stability is mediated by Siah2. EMBO J. 2002;21(21):5756‐5765. 12411493 10.1093/emboj/cdf576 PMC131073

[30] Tracz M , Bialek W . Beyond K48 and K63: non‐canonical protein ubiquitination. Cell Mol Biol Lett. 2021;26(1):1. 33402098 10.1186/s11658-020-00245-6 PMC7786512

[31] Vogel A , Meyer T , Sapisochin G , Salem R , Saborowski A . Hepatocellular carcinoma. Lancet. 2022;400(10360):1345‐1362. 36084663 10.1016/S0140-6736(22)01200-4

[32] Sung H , Ferlay J , Siegel RL , et al. Global cancer statistics 2020: GLOBOCAN estimates of incidence and mortality worldwide for 36 cancers in 185 countries. CA Cancer J Clin. 2021;71(3):209‐249. 33538338 10.3322/caac.21660

[33] Wong VW , Janssen HL . Can we use HCC risk scores to individualize surveillance in chronic hepatitis B infection? J Hepatol. 2015;63(3):722‐732. 26026875 10.1016/j.jhep.2015.05.019

[34] Yang L , Zou T , Chen Y , et al. Hepatitis B virus X protein mediated epigenetic alterations in the pathogenesis of hepatocellular carcinoma. Hepatol Int. 2022;16(4):741‐754. 35648301 10.1007/s12072-022-10351-6

[35] Niu C , Livingston CM , Li L , et al. The Smc5/6 complex restricts HBV when localized to ND10 without inducing an innate immune response and is counteracted by the HBV X protein shortly after infection. PLoS One. 2017;12(1):e0169648. 28095508 10.1371/journal.pone.0169648 PMC5240991

[36] Choi CY , Choi BH , Park GT , Rho HM . Activating transcription factor 2 (ATF2) down‐regulates hepatitis B virus X promoter activity by the competition for the activating protein 1 binding site and the formation of the ATF2‐Jun heterodimer. J Biol Chem. 1997;272(27):16934‐16939. 9202004 10.1074/jbc.272.27.16934

[37] Benn J , Su F , Doria M , Schneider RJ . Hepatitis B virus HBx protein induces transcription factor AP‐1 by activation of extracellular signal‐regulated and c‐Jun N‐terminal mitogen‐activated protein kinases. J Virol. 1996;70(8):4978‐4985. 8764004 10.1128/jvi.70.8.4978-4985.1996 PMC190451

[38] Su F , Schneider RJ . Hepatitis B virus HBx protein activates transcription factor NF‐kappaB by acting on multiple cytoplasmic inhibitors of rel‐related proteins. J Virol. 1996;70(7):4558‐4566. 8676482 10.1128/jvi.70.7.4558-4566.1996 PMC190392

[39] Kim H , Lee H , Yun Y . X‐gene product of hepatitis B virus induces apoptosis in liver cells. J Biol Chem. 1998;273(1):381‐385. 9417092 10.1074/jbc.273.1.381

[40] Liu C , Peng X , Li Y , et al. Positive feedback loop of FAM83A/PI3K/AKT/c‐Jun induces migration, invasion and metastasis in hepatocellular carcinoma. Biomed Pharmacother. 2020;123:109780. 31901550 10.1016/j.biopha.2019.109780

[41] Tsiambas E , Mastronikolis N , Kyrodimos E , et al. c‐Jun/c‐Fos complex in laryngeal squamous cell carcinoma. J BUON. 2020;25(2):618‐620. 32521843

[42] Heaton SM , Borg NA , Dixit VM . Ubiquitin in the activation and attenuation of innate antiviral immunity. J Exp Med. 2016;213(1):1‐13. 26712804 10.1084/jem.20151531 PMC4710203

[43] Miller Z , Ao L , Kim KB , Lee W . Inhibitors of the immunoproteasome: current status and future directions. Curr Pharm Des. 2013;19(22):4140‐4151. 23181576 10.2174/1381612811319220018 PMC3821965

[44] Lin X , Li AM , Li YH , et al. Silencing MYH9 blocks HBx‐induced GSK3β ubiquitination and degradation to inhibit tumor stemness in hepatocellular carcinoma. Signal Transduct Target Ther. 2020;5(1):13. 32296025 10.1038/s41392-020-0111-4 PMC7018736

[45] Song Y , Li M , Wang Y , Zhang H , Wei L , Xu W . E3 ubiquitin ligase TRIM21 restricts hepatitis B virus replication by targeting HBx for proteasomal degradation. Antivir Res. 2021;192:105107. 34097931 10.1016/j.antiviral.2021.105107

[46] Wan T , Lei Z , Tu B , Wang T , Wang J , Huang F . NEDD4 induces K48‐linked degradative ubiquitination of hepatitis B virus X protein and inhibits HBV‐associated HCC progression. Front Oncol. 2021;11:625169. 33767993 10.3389/fonc.2021.625169 PMC7985090

[47] Hu G , Zhang S , Vidal M , Baer JL , Xu T , Fearon ER . Mammalian homologs of seven in absentia regulate DCC via the ubiquitin‐proteasome pathway. Genes Dev. 1997;11(20):2701‐2714. 9334332 10.1101/gad.11.20.2701 PMC316613

[48] Jeong SY , Hariharasudhan G , Kim MJ , et al. SIAH2 regulates DNA end resection and replication fork recovery by promoting CtIP ubiquitination. Nucleic Acids Res. 2022;50(18):10469‐10486. 36155803 10.1093/nar/gkac808 PMC9561274

[49] van Huizen M , Kikkert M . The role of atypical ubiquitin chains in the regulation of the antiviral innate immune response. Front Cell Dev Biol. 2019;7:392. 32039206 10.3389/fcell.2019.00392 PMC6987411

[50] Xu Z , Wu Y , Yang M , Wei H , Pu J . CBX2‐mediated suppression of SIAH2 triggers WNK1 accumulations to promote glycolysis in hepatocellular carcinoma. Exp Cell Res. 2023;426(1):113513. 36780970 10.1016/j.yexcr.2023.113513

