# Supplementary material for: SIAH2 suppresses c‐JUN pathway by promoting the polyubiquitination and degradation of HBx in hepatocellular carcinoma
Source: J Cell Mol Med. 2024 Jun 6;28(11):e18484. doi: 10.1111/jcmm.18484 (PMC11154841; doi:10.1111/jcmm.18484)
Supplement: Supplementary file 1 — Figure S1. [file JCMM-28-e18484-s001.docx]

**Supplemental Figure1**

**
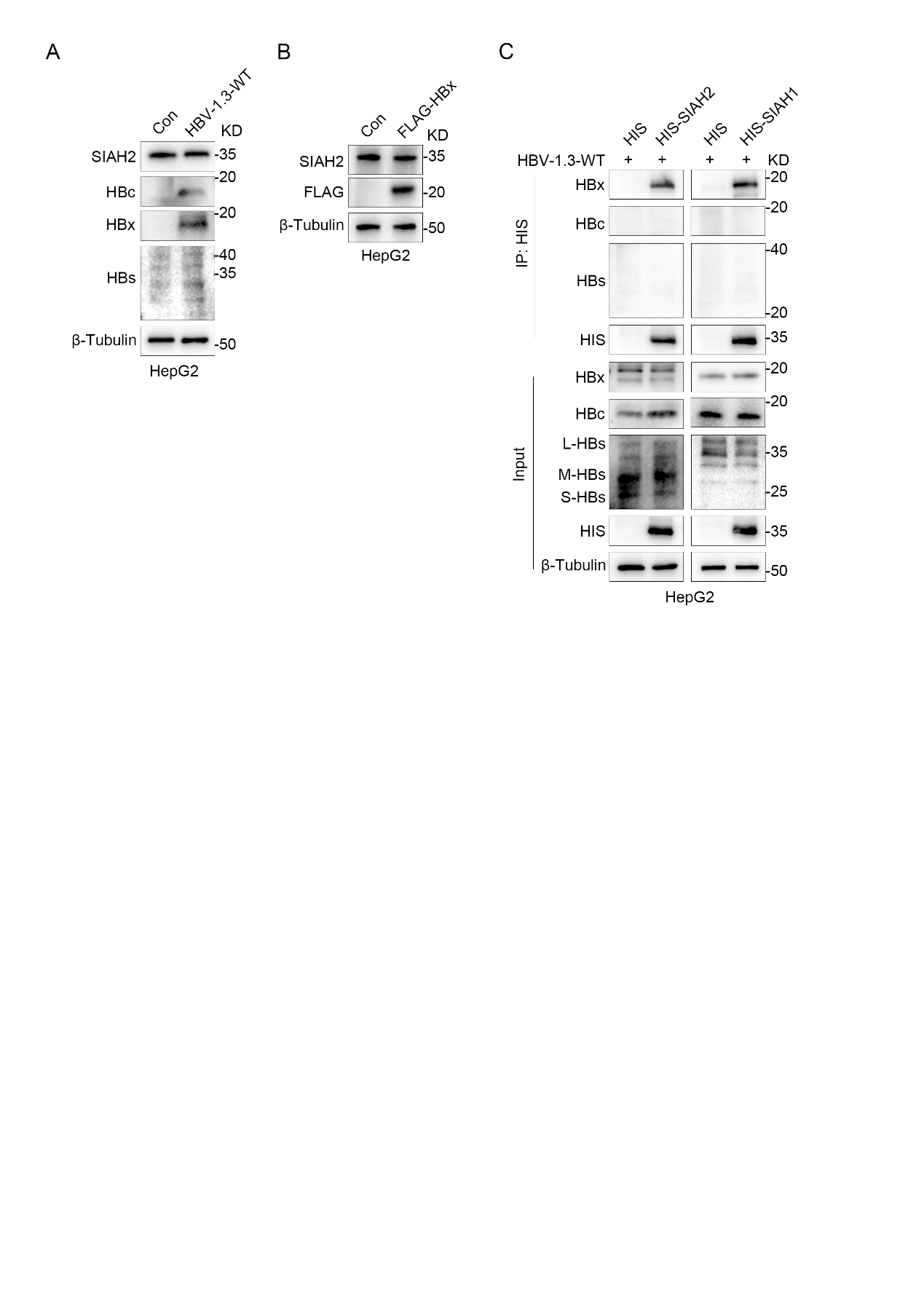
**

**Figure S1. (A)** Transfect the HBV 1.3 WT plasmid in HepG2 cells, and Western blot detected the protein level of SIAH2. **(B)** Transfect FLAG-HBx in HepG2, and Western blot detected the protein level of SIAH2. **(C)** Co-transfect HBV 1.3 WT and SIAH2 or SIAH1 in HepG2, then HIS antibody was used to IP, and Western blot detected the HBc, HBs, and HBx protein.
